# KcsA-Kv1.x chimeras with complete ligand-binding sites provide improved predictivity for screening selective Kv1.x blockers

**DOI:** 10.1016/j.jbc.2024.107155

**Published:** 2024-03-11

**Authors:** Patrik Szekér, Tamás Bodó, Katalin Klima, Ágota Csóti, Nikoletta Ngo Hanh, József Murányi, Anna Hajdara, Tibor Gábor Szántó, György Panyi, Márton Megyeri, Zalán Péterfi, Sándor Farkas, Norbert Gyöngyösi, Péter Hornyák

**Affiliations:** 1 VRG Therapeutics Plc, Budapest, Hungary; 2 Faculty of Medicine, Department of Biophysics and Cell Biology, University of Debrecen, Debrecen, Hungary; 3 Department of Molecular Biology, Semmelweis University, Budapest, Hungary

**Keywords:** Kv1.3 voltage-gated potassium channel, membrane protein, KcsA potassium channel, ion channel, chimeric protein, miniprotein, peptide blocker, inhibitor, peptide toxin, ion channel blocking, phage display, phage ELISA, protein-ligand interaction, protein expression, autoimmune disease

## Abstract

Despite significant advances in the development of therapeutic interventions targeting autoimmune diseases and chronic inflammatory conditions, lack of effective treatment still poses a high unmet need. Modulating chronically activated T cells through the blockade of the Kv1.3 potassium channel is a promising therapeutic approach; however, developing selective Kv1.3 inhibitors is still an arduous task. Phage display-based high throughput peptide library screening is a rapid and robust approach to develop promising drug candidates; however, it requires solid-phase immobilization of target proteins with their binding site preserved. Historically, the KcsA bacterial channel chimera harboring only the turret region of the human Kv1.3 channel was used for screening campaigns. Nevertheless, literature data suggest that binding to this type of chimera does not correlate well with blocking potency on the native Kv1.3 channels. Therefore, we designed and successfully produced advanced KcsA-Kv1.3, KcsA-Kv1.1, and KcsA-Kv1.2 chimeric proteins in which both the turret and part of the filter regions of the human Kv1.x channels were transferred. These T+F (turret-filter) chimeras showed superior peptide ligand-binding predictivity compared to their T-only versions in novel phage ELISA assays. Phage ELISA binding and competition results supported with electrophysiological data confirmed that the filter region of KcsA-Kv1.x is essential for establishing adequate relative affinity order among selected peptide toxins (Vm24 toxin, Hongotoxin-1, Kaliotoxin-1, Maurotoxin, Stichodactyla toxin) and consequently obtaining more reliable selectivity data. These new findings provide a better screening tool for future drug development efforts and offer insight into the target–ligand interactions of these therapeutically relevant ion channels.

Chronic inflammatory conditions and autoimmune diseases affect more than 10% of the world population and cause tremendous suffering to patients. Present therapies are based on nonspecific immunosuppression and consequently, they are associated with numerous side effects. Therefore, there is a huge unmet medical need to develop highly effective new immunomodulators with improved adverse effect profile (1). Chronically activated T cells play a significant role in the pathogenesis of immune inflammation and autoimmunity. The expression of Kv1.3 and KCa3.1 potassium channels in human T cells changes during activation and differentiation. Initially, T cells upregulate KCa3.1 upon antigen activation, but with repeated antigen stimulation, they switch to upregulating Kv1.3. For example, in patients with autoimmune diseases, pathogenic auto-reactive T cells that have been repeatedly stimulated by the relevant autoantigen during the disease exhibit a high expression of Kv1.3. This variation in potassium channel expression between acutely and chronically activated T cells underscores the importance of Kv1.3 as a therapeutic target in chronic inflammatory diseases (2, 3, 4, 5). Therefore, new drugs that block Kv1.3 channels with high potency and high selectivity may open new avenues for the development of more specific immunomodulatory agents with reduced side effects. Currently, there are no Kv1.3 blockers that have reached the market, mainly due to selectivity issues over closely related potassium channels, such as Kv1.1 and Kv1.2 that are highly expressed in neuronal cells (6, 7, 8).

Peptide toxins of venomous animal origin as lead molecules for optimization may offer an advantage over small molecules in terms of potentially greater affinity and specificity. These miniproteins are usually up to 50 amino acids in length and can adopt distinct conformation with secondary structural elements stabilized by several disulfide bonds (9, 10, 11). Due to their considerable surface area, miniproteins can form stable complexes with target proteins through extensive interaction surfaces (12, 13). The miniprotein Kv1.3 blocker drug candidate, Dalazatide—a synthetic derivative of the ShK toxin (isolated from the sea anemone *Stichodactyla helianthus*)—finished phase 1b clinical trials. Dalazatide demonstrated efficacy by significantly reducing plasma levels of multiple inflammation markers. Moreover, in the 60-mcg group, there was a statistically significant reduction in mean PASI score (14, 15). However, Dalazatide also exhibited dose-limiting mild side effects of hypesthesia and paresthesia, which presumably prevented the demonstration of clinically meaningful efficacy. This might be due to the inhibition of Kv1.1 and Kv1.2 ion channels by Dalazatide and/or its metabolite that readily forms upon exposure to human plasma (15). Other peptide toxins with natural origin can also serve as starting point for drug development, such as Vm24 toxin, isolated from the scorpion venom of *Vaejovis mexicanus smithi*. So far, Vm24 is the most potent inhibitor of Kv1.3 with an IC_50_ of 3 pM; however, it has limited selectivity for Kv1.3 over Kv1.2, KCa3.1, and mouse Kv1.1 ion channels (16, 17). In summary, there is a need for highly specific Kv1.3 blockers, and development of miniprotein lead compounds by high-throughput screening (HTS) may be an attractive approach to achieve this goal.

Miniprotein drug candidates can be optimized by HTS technologies, such as phage display, to improve their affinity and selectivity (18, 19). In phage display, the peptide (or miniprotein) is presented on the surface of a bacteriophage, such as M13, in a native conformation. The phage’s phenotype manifests itself by binding to a given target protein through the peptide displayed on its surface, and the genotype can be readily retrieved from the phage’s circular DNA by sequencing. One of the main advantages of the phage display technology is that peptide libraries with millions of variants can be effectively screened in a short time (19). *Via* multiple screening cycles, high affinity binders are enriched in the eluted phage pool and can then be identified by DNA sequencing. This approach has already been used in various screening campaigns to develop potential Kv1.3 blockers (20, 21).

Potassium channels are integral membrane proteins that regulate cellular excitability and maintain ion homeostasis (22). The voltage-gated Kv1.x subfamily plays crucial roles in cellular physiology. Structurally, Kv1.x channels share several conserved features. They form tetrameric complexes, with each subunit comprising six transmembrane helices (denoted S1-S6) and a pore-forming region located between S5 and S6 (23). The S6 segment, together with the P-loop connecting S5 and S6, forms the selectivity filter, which determines the ion selectivity of the channel (Fig. 1*A*). The turret region, protruding into the extracellular environment, is essential for mediating interactions with peptide toxins.

**Figure 1:**
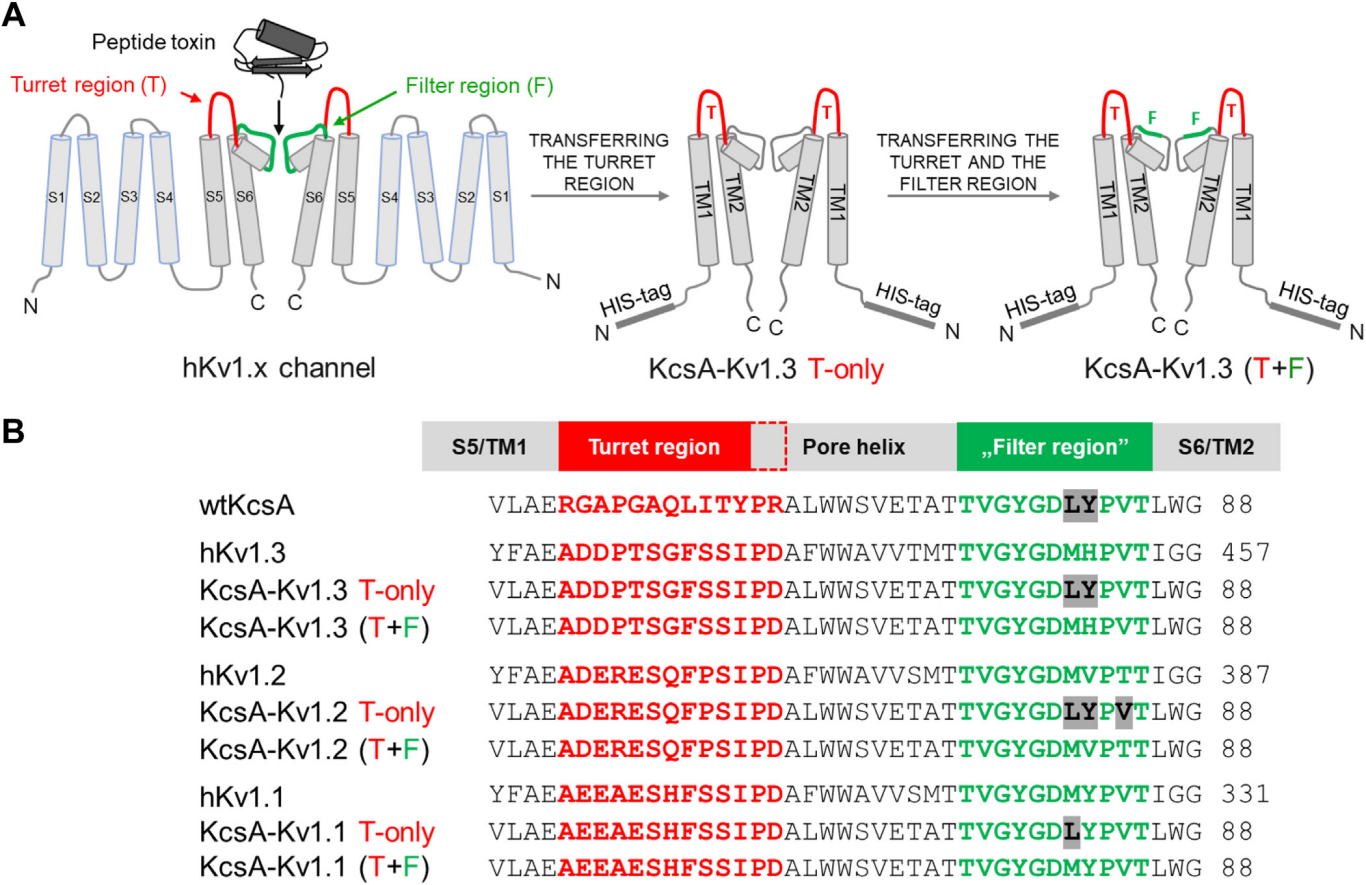
**The subunit structure and sequence of the two distinct versions of the KcsA-Kv1.x chimeras compared with the human Kv1.x channels.***A*, structural characteristics of human Kv1.x (hKv1.x) channel pore domains and their specific regions interacting with pore-blocking peptide toxins are highlighted with *red* (turret region) and *green* (filter region). For clarity, only two opposite subunits of the tetrameric channels are shown for all three channel proteins. For the hKv1.x channel, the six transmembrane helices are denoted from S1 to S6. For the chimeric KcsA-Kv1.x proteins, the two transmembrane helices are denoted as TM1 and TM2. Both the T-only and the T+F chimeras harbor an N-terminal 6× His-tag. In the KcsA-Kv1.x T-only chimera, the position of transferred turret region is highlighted with *red*, and in the T+F mutant, the mutated regions of the turret and filter are highlighted with *red* and *green*, respectively. *B*, comparative list of the amino acid composition of the turret and filter regions of the WT KcsA, the human Kv1.1-3 proteins and the turret-only and the turret-filter chimeras. The number after each sequence indicates the position of the last amino acid presented. The transferred turret and filter regions are highlighted in *red* and *green*, respectively. Amino acid differences in the filter region between the native protein, turret-filter, and turret-only chimeras are highlighted in *gray*. C, C-terminal; F, filter; N, N-terminal; T, turret.

The Kv1.3 potassium channel is highly expressed in immune cells and plays a crucial role in the regulation of their functions, such as controlling membrane potential and calcium signaling. Thereby, they affect signal transduction pathways regulating the activation and proliferation of T cells following antigenic stimulation (24). Dysregulation of Kv1.3 has been associated with various autoimmune disorders, such as multiple sclerosis, rheumatoid arthritis, and psoriasis (25). As stated above, selective blocking of Kv1.3 may allow for the suppression of the activity of pathogenic immune cells involved in chronic inflammatory diseases, while leaving protective immune functions intact. Thus, inhibition of the Kv1.3 channel might provide a more targeted and potentially safer approach compared to broad immunosuppression (26, 27, 28).

The significant but limited sequence diversity in the turret and filter regions of Kv1.x subtypes poses a huge challenge in creating highly selective Kv1.3 blockers (29). Moreover, utilization of recombinantly expressed human Kv1.3 channels in solid-phase screening campaigns is extremely cumbersome due to the poor stability of tetramers in a detergent-only environment (30). Cell-based screening of drug libraries and potential candidates measuring the activity of membrane-embedded potassium channels such as thallium flux assay (31) and automated patch clamp (32) are promising approaches, but they are still unable to screen peptide libraries containing millions of variants in a short time and at a reasonable cost. During the last 2 decades, the bacterial KcsA channel (K channel of *streptomyces* A), isolated from *Streptomyces lividans*, emerged as a potential model system to study structure, function, and target–ligand interactions of Kv1.x channels due to its relatively simple production in bacterial recombinant expression systems (33). This potassium ion channel is composed of a homotetrameric complex, with each subunit containing two transmembrane helices (TM1 and TM2) connected by a pore loop. The KcsA channel exhibits structural similarities with the pore domain (S5–S6) of the Kv1.x channels (Fig. 1). Building on these resemblances, Legros *et al.* (34) demonstrated that a hybrid construct in which the turret region of the human Kv1.3 was transferred to the homologous part of KcsA protein can be expressed recombinantly, and this chimeric protein (denoted KcsA-Kv1.3 turret-only, KcsA-Kv1.3 T-only for short) is able to bind radiolabeled peptide toxins targeting the native Kv1.3. This chimera was used in a HTS campaign that resulted in a peptide with high binding affinity to Kv1.3 (20, 30). Following the production of the KcsA-Kv1.3 T-only chimeric construct, other members of the Kv1.x subfamily were also expressed in T-only chimeric forms (35). However, several studies have shown that in addition to the turret region, part of the filter region is also responsible for selective ligand binding, therefore the validity of the T-only chimeras to predict relative binding affinities of potential ligand molecules might be limited (16, 36, 37, 38, 39, 40, 41, 42, 43). Attempts to recombinantly produce active chimeras harboring both the turret and the filter region (denoted as T+F) of human Kv1.x channels in heterologous expression systems were unsuccessful due to poor expression and inaccurate assembly of monomers resulting in defective tetramers (34, 44).

As it was stated above, the validity of the T-only chimeras might be insufficient; therefore, in the present study, we conducted experiments to characterize relative affinities of a set of peptide toxins towards the KcsA-Kv1.3 T-only chimera and compared the results with published potencies obtained with the native human Kv1.3 channel. Our results showed that the T-only chimeras are not suitable for accurately recapitulating the relative channel blocking potencies of the tested toxins on the human Kv1.3. This led us to produce T+F chimeras not only for KcsA-Kv1.3 but also for Kv1.1 and 1.2 channels (see Fig. 1), which enabled us to study how potassium channel blocker toxins bind to both T+F and T-only chimeric versions of Kv1.x channels. We investigated the binding of peptide toxins both in phage-displayed and chemically synthesized test substance form, employing phage ELISA and competition tests, respectively. The tested set of peptide toxins comprised the following: Vm24, Hongotoxin-1 (HgTx1; originally isolated from the scorpion venom of *Centruroides limbatus*), Kaliotoxin-1 (KTX1; from the scorpion *Androctonus mauretanicus mauretanicus*), Maurotoxin (MTX; from *Scorpio maurus palmatus*), and ShK.

Our findings contribute to improve the solid-phase HTS further in the development of miniprotein-based drugs for Kv1.x channels and moreover, to provide *in vitro* biochemical data about the structural basis of toxin selectivity over Kv1. 1, Kv1. 2, and Kv1. 3 channels.

## Results

### Expression and purification of KcsA-Kv1.x T-only and T+F chimeras

**Figure 2:**
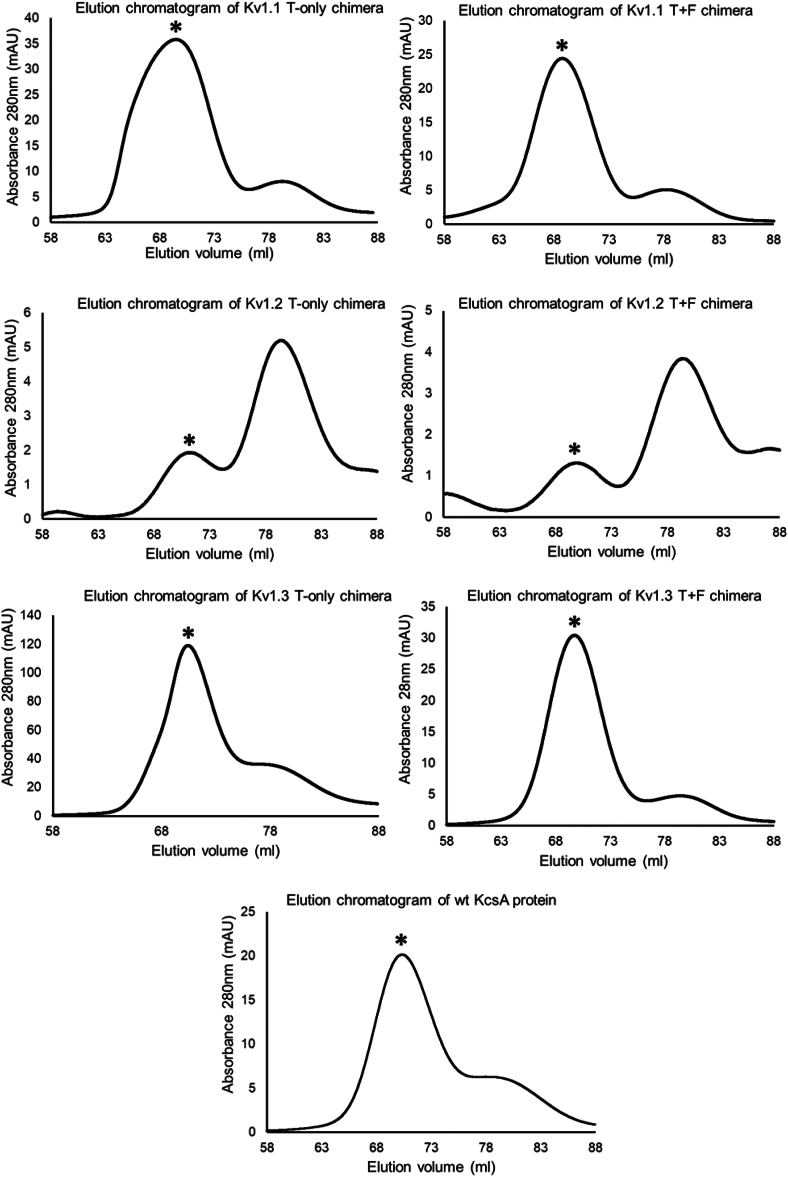
**Elution profile of KcsA-Kv1.x chimeras in gel filtration chromatography.** Representative gel filtration chromatograms of WT KcsA and KcsA-Kv1.x T-only and T+F chimeras. Protein samples were loaded onto HiLoad 16/600 Superdex 200 pg gel filtration column (Cytiva) and eluted at 1 ml/min flow rate. In each chromatogram, elution peak containing the active tetrameric chimeric proteins is marked with a *black asterisk* (∗). T+F, Turret-Filter mutant KcsA Kv1.x chimera.

**Figure 3:**
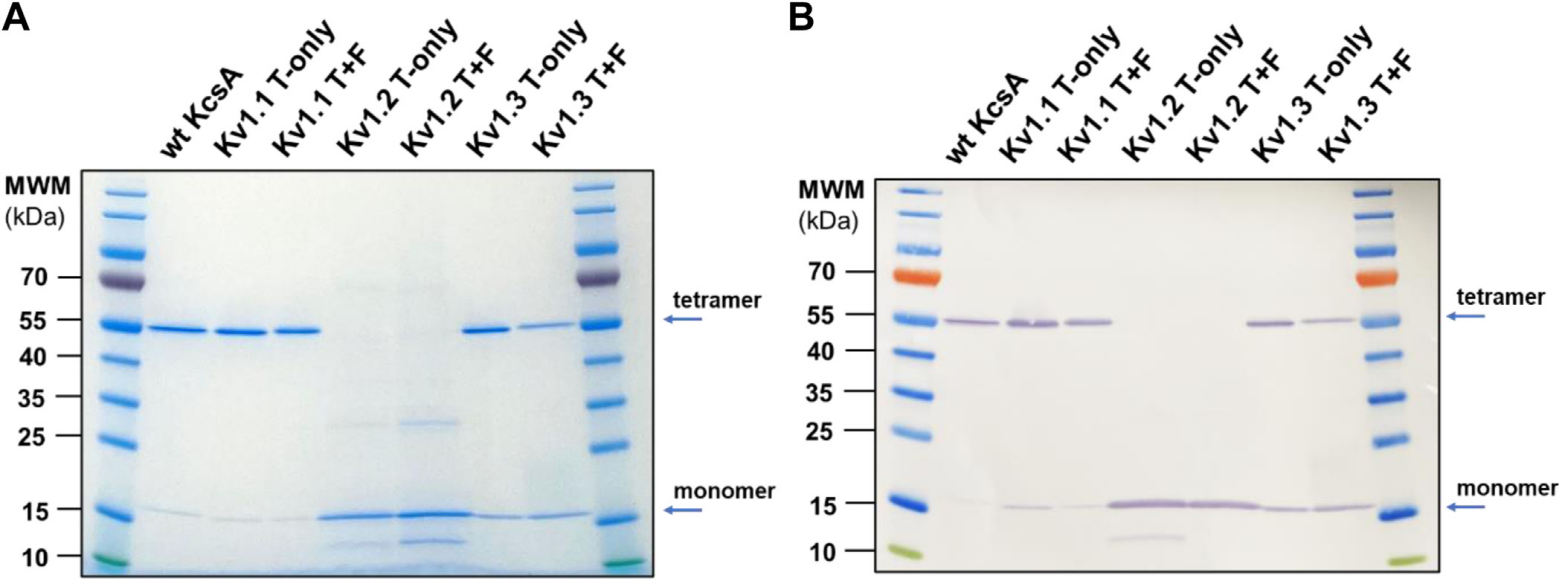
**SDS-PAGE and western blot analysis of KcsA-Kv1.x chimeras.***A*, SDS-PAGE analysis of purified KcsA-Kv1.x chimeras. Eluted fractions from the gel filtration step containing the active tetrameric chimeric proteins (see in Fig. 2.) were pooled and concentrated, then stored in aliquots at −80 °C. One aliquot/chimera (0.5 μg sample/well) was loaded onto a Tris-Glycine 4 to 16% gradient gel (Thermo Fisher Scientific). Running conditions: 200 V, 55 min. Gel was stained with ReadyBlue Protein Gel Stain (RSB-1L, Sigma Aldrich). *B*, western blot analysis of purified KcsA-Kv1.x chimeras (0.5 μg sample/well). Blotting conditions: 25 V for 120 min. Chimeras were detected with mouse 6×-His tag monoclonal primary antibody (4E3D10H2/E3, Invitrogen) and Goat anti-mouse IgG HRP-conjugated secondary antibody (31430, Invitrogen). Blotting solution: Pierce 1-Step Ultra TMB Blotting Solution (Thermo Fisher Scientific). T+F, Turret-Filter mutant of KcsA-Kv1.x chimera.

**Figure 4:**
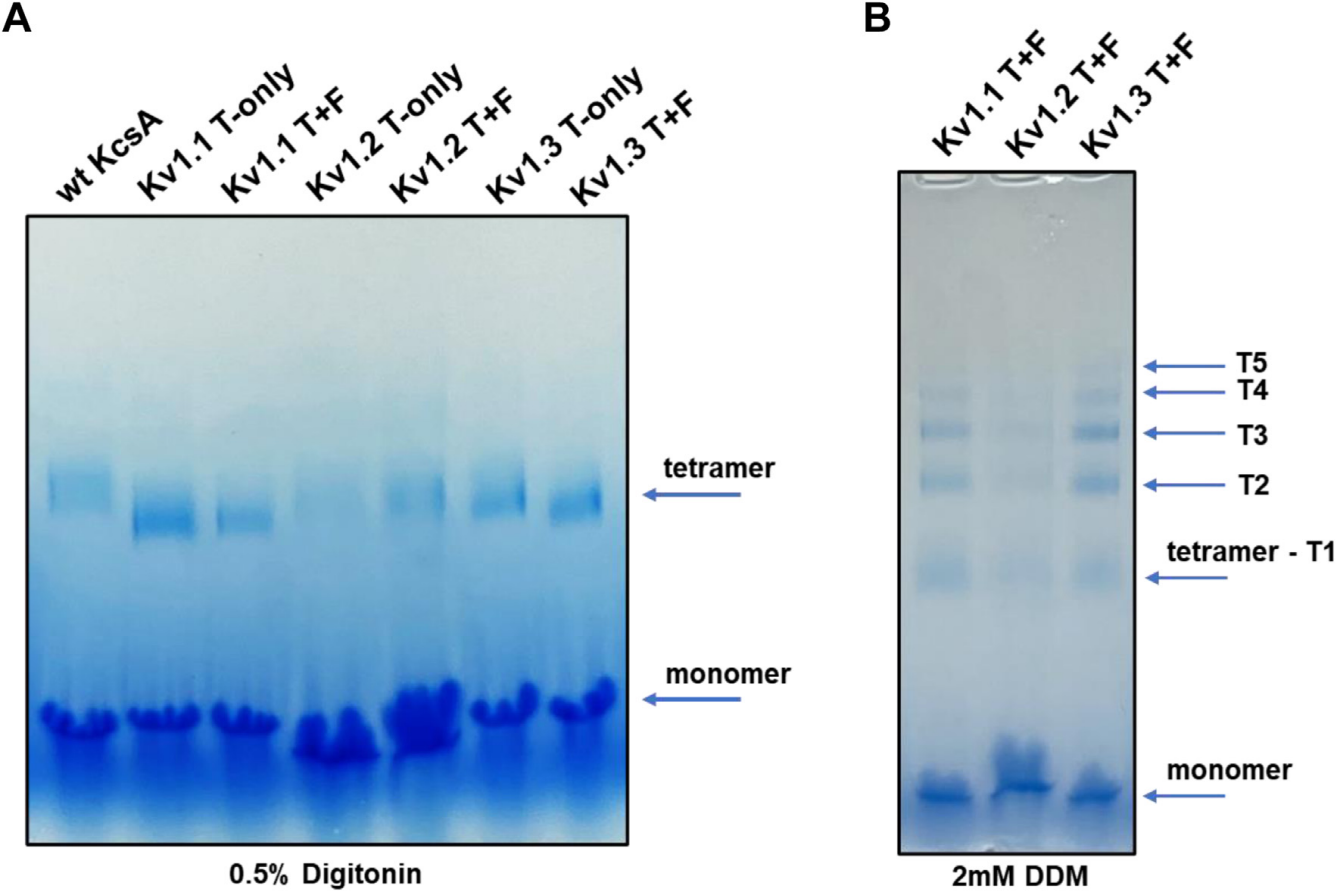
**Native PAGE analysis of KcsA-Kv1.x chimeras.** Native PAGE analysis of purified KcsA-Kv1.x chimeras. 0.5 μg sample/well was loaded onto 4 to 16% gradient Bis-Tris gel (Invitrogen) supplemented with the indicated concentrations of Digitonin (*A*) or N-Dodecyl-β-D-maltoside (DDM, *B*) detergents. Running conditions: 150 V, 105 min. Due to experimental conditions, up to n = 5 species can be observed. T1, single tetramer; T2-T5, multimers of tetrameric chimeras; T+F, Turret-Filter mutant KcsA-Kv1.x chimera.

We sought to generate turret-filter (T+F) chimeric versions of our target channels and compare their peptide toxin binding profile with turret-only (T-only) chimeras in a solid-phase assay format. The DNA constructs coding the chimeric proteins were cloned into pQE or pET expression plasmids (See Experimental procedures). To obtain substantial amounts of active proteins (it is important to emphasize that “active” refers to the tetramer’s ability to bind peptide blockers, or peptides displayed on the surface of M13 bacteriophages), we established new and improved expression and purification protocols by adjusting several parameters (see Experimental procedures for details). Among the crucial factors influencing the production of active protein, the choice of the appropriate expression strain proved to be very important. The C41 (DE3) *Escherichia coli* strain, designed to produce difficult to express or toxic proteins (43), proved to be the most effective. Preliminary expression attempts revealed that KcsA-Kv1.1, KcsA-Kv1.2, and KcsA-Kv1.3 T+F and T-only chimeric proteins could be expressed in the membrane fractions of various *E. coli* expression strains. However, only the C41 strain produced considerable amounts of active tetramers that exerted peptide-binding capability. The expressed proteins were purified through a series of steps, including high-speed centrifugation to isolate the membrane fraction of lysed bacteria, followed by two chromatographic steps: immobilized metal affinity chromatography and gel filtration chromatography. The chromatogram of the gel filtration showed several elution peaks for each chimeric protein (Fig. 2). Elution peaks containing active tetramers were identified by phage ELISA (see Experimental procedures for details). Fractions containing active tetramers were pooled, concentrated, then aliquoted, and stored at −80 °C. Purified proteins were analyzed by SDS-PAGE. The results clearly indicate that all chimeric and the WT KcsA proteins were of great purity (Fig. 3*A*). In addition, both monomeric and tetrameric forms were identified by employing western blot using mouse anti-His monoclonal antibody against the N-terminal 6xHis-tag fused to the chimeric proteins. Monomers and tetramers migrated at the expected molecular weight indicating intact proteins (Fig. 3*B*). Although recombinantly expressed KcsA preserves its tetrameric structure during SDS-PAGE analysis (45), neither the T-only nor the T+F KcsA-Kv1.2 chimeras shown detectable level of the tetramers in SDS-PAGE analysis, suggesting that these proteins were more sensitive to denaturing and reducing conditions. To show the presence of tetramers, we also analyzed our chimeric proteins in native PAGE. Most membrane proteins require detergent-containing loading buffer when running under native conditions; therefore, we tested the purified chimeras in the presence of the mild non-ionic detergents digitonin and N-Dodecyl-β-D-maltoside (DDM). We were able to identify the tetrameric forms of all chimeras in the condition where digitonin was used as detergent (Fig. 4*A*). The KcsA-Kv1.1, KcsA-Kv1.2, and KcsA-Kv1.3 T+F chimeras were also investigated in the presence of DDM by native PAGE. Under this condition, we identified tetramers in their multimeric forms as well (Fig. 4*B*). This observation aligns with the findings of previous studies investigating the supramolecular assemblies of the KcsA channel protein in the presence of various detergents (46).

### Assessment of predictivity of the KcsA-Kv1.3 chimera

**Figure 5:**
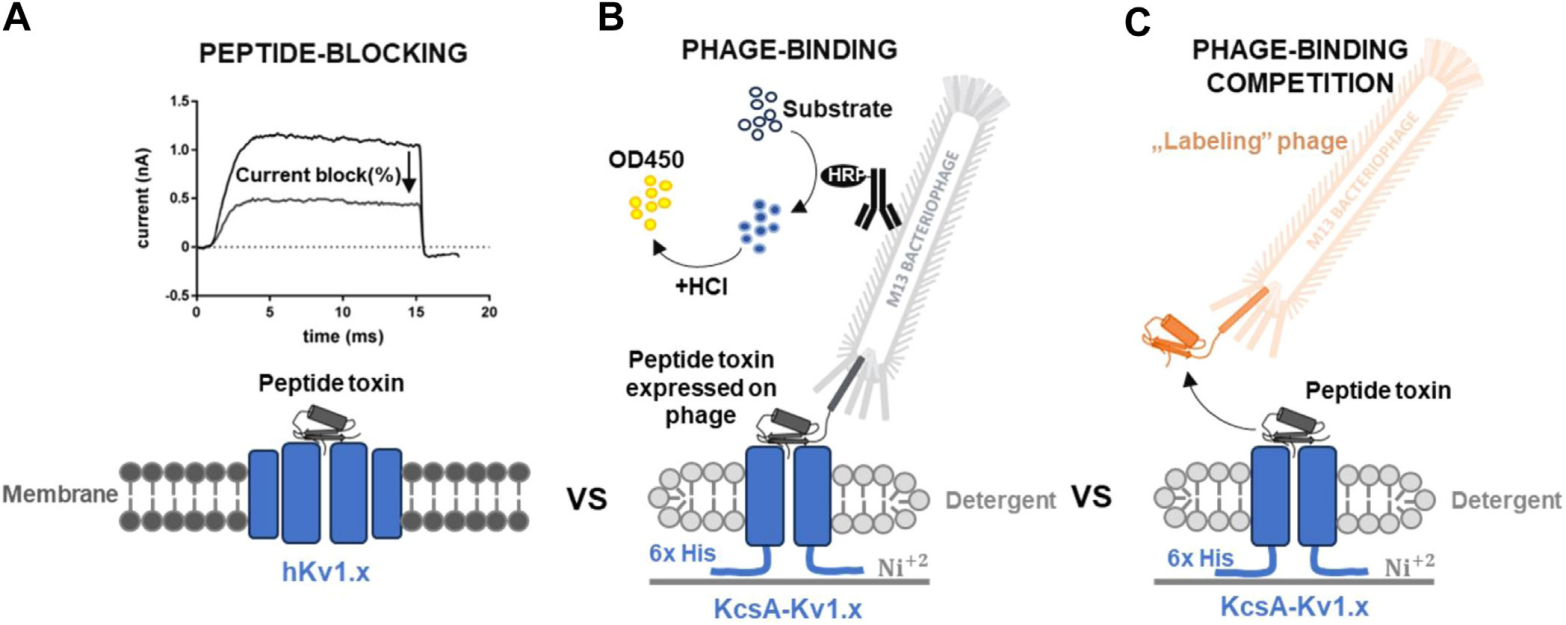
**The illustration of the comparative analysis of KcsA-Kv1.x T-only and T+F chimeric proteins with human Kv1.x channel proteins.***A*, peptide-blocking: human Kv1.x channel (hKv1.x) blocking of toxin test substances was investigated in whole-cell patch-clamp experiments. Whole-cell current traces were recorded using the appropriate voltage protocol for each hKv1.x channel before and after perfusion administration of the tested toxin. *B*, phage-binding test: The method involved immobilizing a His-tagged chimeric target protein on a nickel-coated plate, adding toxin peptide displaying phages that bind to the target, washing away unbound particles, labeling with the HRP-conjugated antibody, and detecting the signal produced by HRP catalysis upon substrate addition. *C*, phage-binding competition test: Binding of given concentrations of labeling (HgTX1- or ShK- displaying) phages was investigated in a similar manner as in (*B*), while different concentrations of competing toxin test substances were applied simultaneously with the labeling phages.

Figure 5 depicts the methods we used to assess the binding and blocking potency of the selected peptide toxin-displaying phages and the synthesized miniproteins. To confirm pharmacological rank order, we measured the blocking potency of selected toxins on Kv1.1, Kv1.2, and Kv1.3 channels in human T cells in whole-cell patch-clamp experiments (Figs. 5*A*, and S1–S3). To assess the relative binding affinities of the compounds, phage ELISA was used to detect the binding of peptide toxin-displaying phages to Kv1.x chimera-coated surfaces (Fig. 5*B*). In addition, we characterized the displacing potency (IC_50_) of the synthetic peptide toxins (Fig. 5*C*). In this competition test, we used phages displaying HgTx1 for Kv1.3 and Kv1.2 and ShK for Kv1.1, as indicator ligands to be displaced by synthetic peptide toxins.

Our initial investigation focused on assessing the relative binding affinities of various channel-blocking toxins to the T-only version of the KcsA-Kv1.3 chimera. Subsequently, we evaluated whether these affinities aligned with the toxins' blocking efficacy on native human Kv1.3 channels, as determined by patch-clamp assay. For this purpose, we selected three toxins with different Kv1.3 channel–blocking potencies: Vm24, Hongotoxin-1 (HgTx1), and Kaliotoxin-1 (KTX1) with reported IC_50_ values of 3 pM, 86 pM, and 650 pM in patch-clamp experiments, respectively (16, 47, 48) (Table 1).

**Table 1:** Summary of chimeric protein-binding prediction studies in the light of IC_50_ values

Toxin ligand	T-only chimera		T+F chimera		Kv1.x blocking		
	IC_50_ (nM)	Rank	IC_50_ (nM)	Rank	IC_50_ (nM)	Rank	References
Kv1.3							
Vm24	0.577 (±0.431)	2	0.036 (±0.004)	1	0.003	1	(16)
HgTx1	0.671 (±0.090)	3	0.108 (±0.022)	2	0.086	2	(47)
KTX1	0.416 (±0.062)	1	98.2 (±81.6)	3	0.650	3	(48)
Kv1.2							
MTX	>1000 (NQ)	3	0.129 (±0.050)	1	0.8	1	(36)
ShK	0.070 (±0.026)	1	15.9 (±4.4)	2	9.0	2	(49)
KTX1	0.185 (±0.031)	2	205.3 (±59.4)	3	>1000	3	(48)
Kv1.1							
ShK	0.317 (±0.069)	1	0.271 (±0.037)	1	0.007–0.09	1	(49)
KTX1	1.270 (±0.308)	2	1.487 (±0.260)	2	1.1–41	2	(48)
MTX	>1000 (NQ)	3	>1000 (NQ)	3	>100	3	(36)

IC_50_ values for KcsA-Kv1.x T-only and T+F chimeras were obtained in phage competition assay; human Kv1.x channel blocking literature data obtained by patch-clamp assay are presented. Values are presented as mean ± SD (n = 3). NQ stands for not quantifiable.

**Figure 6:**
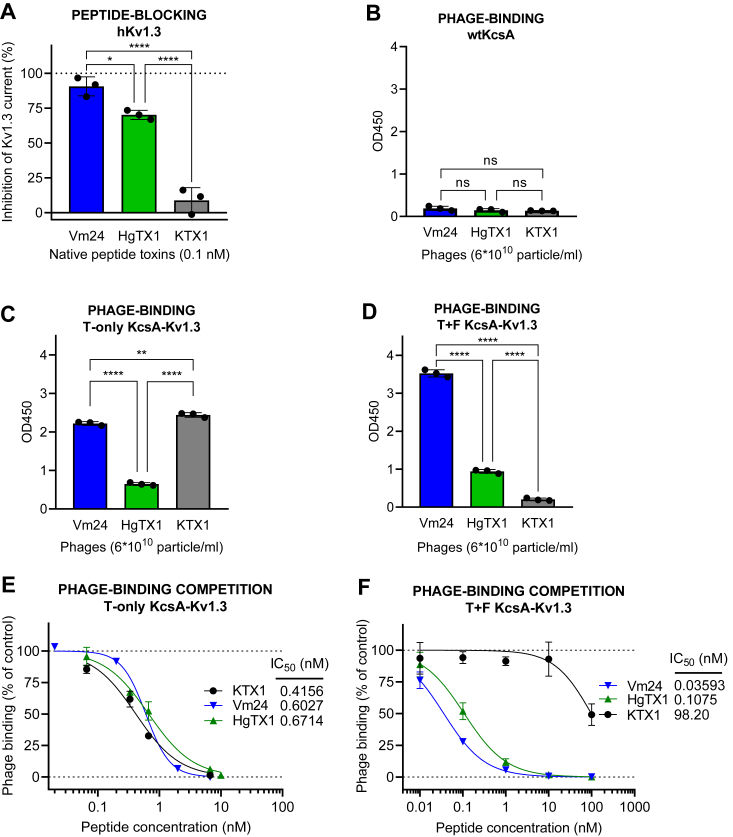
**Comparison of KcsA-Kv1.3 T-only and T+F chimera binding to native Kv1.3 channel blocking potency.***A*, blocking effect (percent inhibition of membrane potassium current) of peptide toxins Vm24, HgTX1, and KTX1 on the human Kv1.3 channel (hKv1.3) at a concentration of 0.1 nM, as measured by patch-clamp on human lymphocytes. *B*, binding of toxin-expressing phages to WT KcsA (negative control), as measured by absorbance in phage ELISA assay. *C*, binding of phages expressing the three toxins to KcsA-Kv1.3 T-only in the phage-binding test. *D*, binding of phages expressing the three toxins to T+F KcsA-Kv1.3 in the phage-binding test. *E*, percent phage binding *versus* log concentration plots and IC_50_ values calculated for the toxin test substances, obtained in the HgTx1-phage binding competition assay carried out on T-only KcsA-Kv1.3–coated plates, as measured by absorbance in phage ELISA assay. *F*, percent phage binding *versus* log concentration plots and IC_50_ values calculated for the toxin test substances, obtained in the HgTx1-phage binding competition assay carried out on T+F KcsA-Kv1.3–coated plates. The bar graphs are presented as means ± SD of three replicates with superimposed scatter plots. The phage binding *versus* log concentration plots also represent means ± SD of three replicates with sigmoidal curve fitting. Differences between all three IC_50_ values in (*F*) (but not in *E*) were statistically significant. A one-way ANOVA and post hoc Tukey HSD tests confirmed significant differences between group means, as marked on the figures; nonsignificant results are labeled ‘ns’. HgTX1, Hongotoxin-1; KTX1, Kaliotoxin-1; Vm24, Vm24 toxin.

Comparing the efficacy of the chosen toxins to block the native Kv1.3 channel during patch-clamp experiments showed striking differences from the results of binding assays to the T-only chimeric channel (Fig. 6, *A*–*E*). Whereas electrophysiological blocking efficiencies were consistent with published potencies, exhibiting a rank order of Vm24 > HgTX1 > KTX1 (Figs. 6*A* and S1, and Table 1), the phage-binding results suggested similar binding efficiencies for Vm24-and KTX1- and a weaker binding of HgTX1-phage to the T-only chimera (Fig. 6*B*). Affinities of the three toxins to the T-only chimeras in the competition binding test were similar with IC_50_ values ranging from 0.42 to 0.67 nM (Fig. 6*E*). As shown in Figure 6*B*, none of the tested phage-displayed toxins showed nonspecific binding to the WT KcsA.

Since KcsA-Kv1.3 T-only chimeras apparently did not exhibit good predictive power for Kv1.3 channel blocking potency, we investigated the binding of the selected toxins to the KcsA-Kv1.3 T+F chimeras as well (Fig. 6, *D* and *F*). We found that rank order of affinities both in phage-binding (Fig. 6*D*) and competition assays (Fig. 6*F*) were consistent with that of blocking potencies on native Kv1.3 channels in our patch-clamp studies (Vm24 > HgTx1 > KTX1), showing that KcsA-Kv1.3 T+F chimera is more reliable than KcsA-Kv1.3 T-only.

### Assessment of predictivity of KcsA-Kv1.1 and KcsA-Kv1.2 chimeras

**Figure 7:**
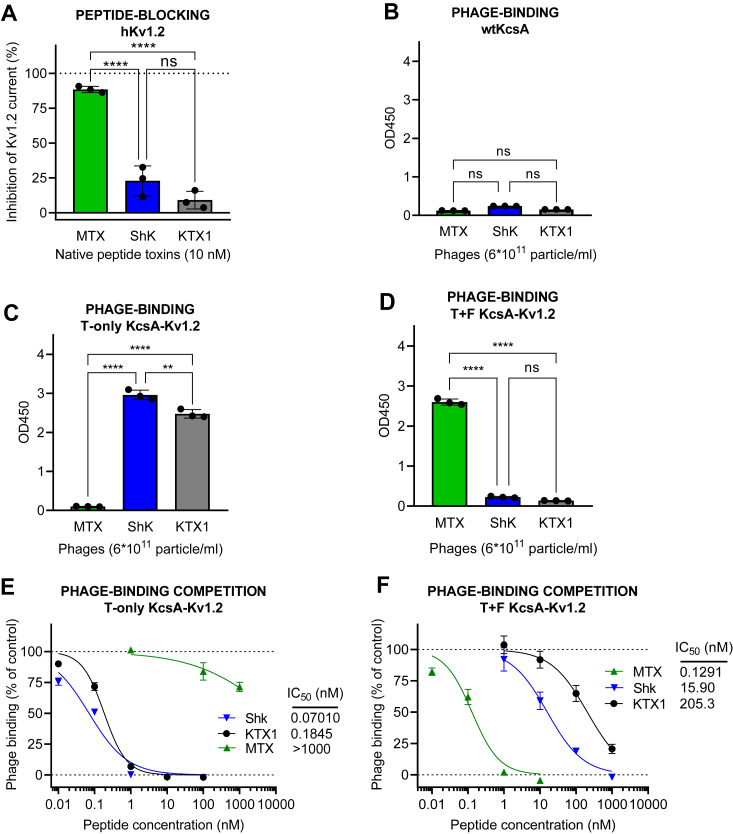
**Comparison of KcsA-Kv1.2 T-only and T+F chimeras binding to native human Kv1.2 channel blocking potency.***A*, blocking effect (percent inhibition of membrane potassium current) of peptide toxins MTX, ShK, and KTX1 on the human Kv1.2 channel (hKv1.2) at a concentration of 10 nM, as measured by patch-clamp on hKv1.2-expressing CHO cells. *B*, binding of phages expressing the three toxins to WT KcsA (negative control) in the phage-binding test, as measured by absorbance in phage ELISA assay. *C*, binding of phages expressing the three toxins to KcsA-Kv1.2 T-only in the phage-binding test. *D*, binding of phages expressing the three toxins to KcsA-Kv1.2 T+F in the phage-binding test. *E*, percent phage binding *versus* log concentration plots and IC_50_ values calculated for the toxin test substances, obtained in the HgTx1-phage binding competition assay carried out on KcsA-Kv1.2 T-only–coated plates, as measured by absorbance in phage ELISA assay. *F*, percent phage binding *versus* log concentration plots and IC_50_ values calculated for the toxin test substances, obtained in the HgTx1-phage binding competition assay carried out on KcsA-Kv1.2 T+F-coated plates. The bar graphs are presented as means ± SD of three replicates with superimposed scatter plots. The phage binding *versus* log concentration plots also represent means ± SD of three replicates with sigmoidal curve fitting. Differences between all three IC_50_ values in (*E*) and (*F*) were statistically significant. A one-way ANOVA and post hoc Tukey HSD tests confirmed significant differences between group means, as marked on the figures; nonsignificant results are labeled ‘ns’.

In case of the human Kv1.2 and Kv1.1 channels, the selected test toxins for comparative studies were MTX, ShK, and KTX1, since their potency values span a wide range from sub-nanomolar to 100 nanomolar scale. Their potency ranking is MTX > ShK >> KTX1 for Kv1.2 and ShK > KTX1 >> MTX for Kv1.1 (see Table 1 and Figs. S2 and S3). Binding of the aforementioned toxins to KcsA-Kv1.2 T-only showed significant discrepancies in their potencies to block native Kv1.2 channel. MTX1, a potent blocker of the native Kv1.2 channel (see Fig. 7*A* and Table 1) (36, 42), exhibited negligible binding to the KcsA-Kv1.2 T-only chimera either in phage-binding (Fig. 7*C*) or competition assay (Fig. 7*E*). In parallel, rank order of the affinities of ShK and KTX1 were inconsistent with their published channel blocking potencies as well (Fig. 7, *C* and *E* and Table 1) (48, 49). In contrast, the rank order of affinities of the three tested toxins was in accordance with their channel blocking potencies in case of KcsA-Kv1.2 T+F chimera (Fig. 7, *A*, *D*, and *F* and Table 1).

**Figure 8:**
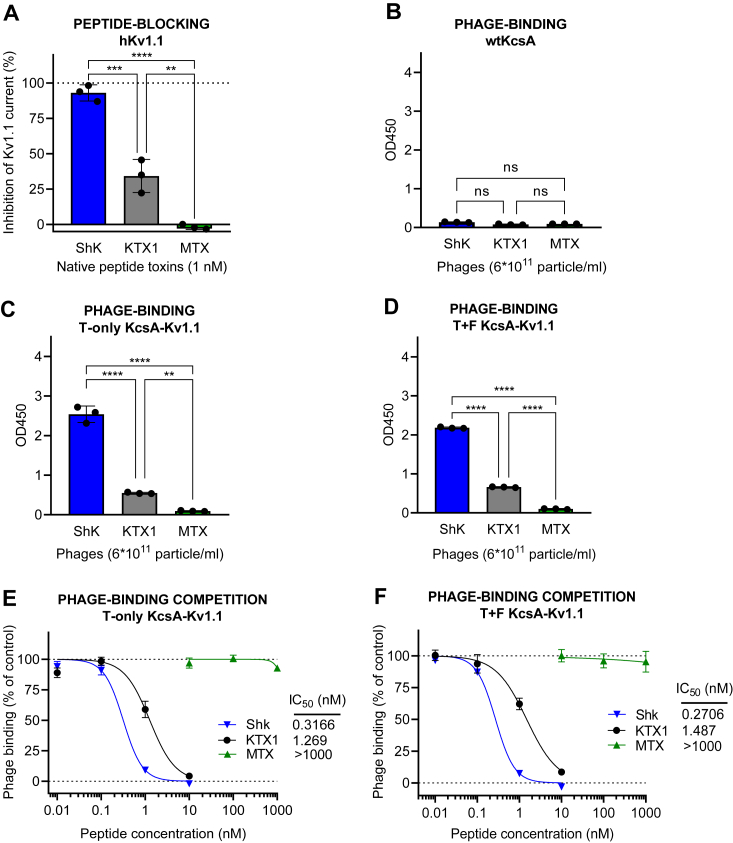
**Comparison of KcsA-Kv1.1 T-only and T+F chimera binding to native Kv1.1 channel blocking potency.***A*, blocking effect (percent inhibition of membrane potassium current) of peptide toxins ShK, KTX1, and MTX on the human Kv1.1 channel (hKv1.1) at a concentration of 1 nM, as measured by patch-clamp on hKv1.1-expressing CHO cells. *B*, binding of phages expressing the three toxins to WT KcsA (negative control) in the phage-binding test, as measured by absorbance in phage ELISA assay. *C*, binding of phages expressing the three toxins to T-only KcsA-Kv1.1 in the phage-binding test. *D*, binding of phages expressing the three toxins to T+F KcsA-Kv1.1 in the phage-binding test. *E*, percent phage binding *versus* log concentration plots and IC_50_ values calculated for the peptide toxins, obtained in the ShK-phage binding competition assay carried out on T-only KcsA-Kv1.1–coated plates, as measured by absorbance in phage ELISA assay. *F*, percent phage binding *versus* log concentration plots and IC_50_ values calculated for the peptide toxins, obtained in the ShK-phage binding competition assay carried out on T+F KcsA-Kv1.1–coated plates. The bar graphs are presented as means ± SD of three replicates with superimposed scatter plots. The phage binding *versus* log concentration plots also represent means ± SD of three replicates with sigmoidal curve fitting. Differences between all three IC_50_ values in (*E*) and (*F*) were statistically significant. A one-way ANOVA and post hoc Tukey HSD tests confirmed significant differences between group means, as marked on the figures; nonsignificant results are labeled ‘ns’.

Predictivity of KcsA-Kv1.1 chimera ligand binding for human Kv1.1 channel blocking potency was assessed in a similar manner as shown above for the other KcsA-Kv1.x variants. However, in the competition-binding assay, ShK-phage was used as the displaced indicator ligand due to the fact that ShK-phage exhibits exceptionally high affinity for the Kv1.1 receptor, making the assay more sensitive than using HgTX1-phage. The increased sensitivity provided by ShK-phage allowed for a more precise assessment of binding interactions with the KcsA-Kv1.1 chimeras. Contrary to KcsA-Kv1.2 and -Kv1.3 chimeras, the phage binding and competition binding results (Fig. 8, *C*–*F*) were very similar for both KcsA-Kv1.1 chimera versions (T-only and T+F), indicating similar affinities of the test toxins to both versions with a rank order of ShK > KTX1 and negligible binding of MTX1, either in phage-displayed (Fig. 8, *C* and *D*) or in native peptide form (Fig. 8, *E* and *F*). Thus, compared to the native hKv1.1 channel–blocking potency rankings (Fig. 8*A* and Table 1), both the T-only and the T+F chimeras appeared to provide good predictions for the native Kv1.1. The close predictive value of the two KcsA-Kv1.1 chimeras is the consequence of their similar protein sequence (only one amino acid difference) in contrast to the KcsA-Kv1.2 and KcsA-Kv1.3 chimeras that differ in two and three residues in the filter region, respectively (see Fig. 1).

The results of comparative predictivity assessments are summarized in Table 1, where affinities of the tested toxins are represented by IC_50_ values derived from the competition assays and compared to reported channel-blocking potencies on the corresponding native hKv1.x channels.

In summary, our results indicate that binding of toxins to the T+F version of the KcsA-Kv1.x chimeras are in good correlation with electrophysiological blocking potencies on Kv1.x channel These data support the superiority of our T+F variants to screen toxin-phage libraries in contrast to the T-only version.

### Utility of toxin expressing phages for functional assessment

**Figure 9:**
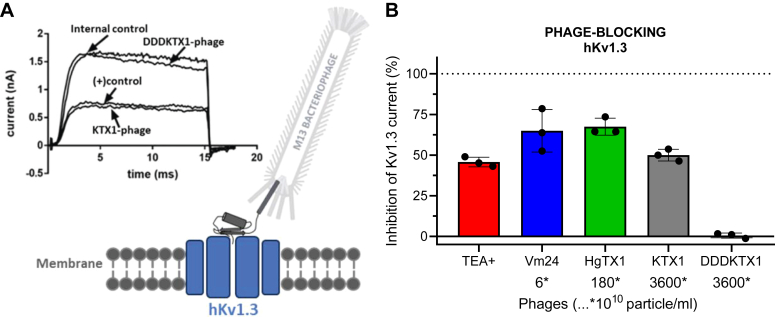
**Human Kv1.3 potassium channel blocking effects of phage-displayed toxin peptides.***A*, illustration depicting a peptide toxin expressed on a phage and binding to a human Kv1.3 channel protein (hKv1.3) and representative whole-cell current traces with tetraethylammonium (TEA+, 10 mM) as positive control, KTX1-phage, and DDDKTX1-phage. The peptide toxins were expressed as fusions to the P3 coat protein of M13 phages, with an eight amino acid long linker region (see Experimental procedures for details). *B*, blocking effect (percent inhibition of membrane potassium current) of phage-expressed peptide toxins on the hKv1.3 channel at different “concentrations”, as measured by patch-clamp on human lymphocytes. After recording control traces, the rapidly acting reversible potassium channel blocker TEA+ at 10 mM was applied as positive control substance (*red*), which usually provides approximately 50% inhibition on hKv1.3 currents. After washing out TEA+ and recovery of the control current, KTX1 displaying phages were applied at a perfusion concentration of 3.6 × 10^13^ particle/ml (*gray*). Following wash out, the inactive mutant DDDKTX1-phages (*black*) were applied at the same concentration and in contrast with KTX1 were apparently ineffective. The bar graph shows the current blocking effects of HgTX1 (*green*) and Vm24 toxin (*blue*) expressing phages at 1.8 × 10^12^ particle/ml and 6 x 10^10^ particle/ml, respectively. The results are presented as mean ± SD of peak currents (N = 3 each). Note the differences in the concentrations of the different but similarly effective (50–70% inhibitory) toxin-expressing phages, which are in line with differences in the Kv1.3-blocking potencies of these peptides.

To bridge the gap between binding and blocking data, we conducted a complementary experiment where the toxins were tested in a phage-displayed form. This experiment focused on assessing the human Kv1.3 channel blocking efficiency of three selected test toxins (Vm24, HgTX1, KTX1) in phage-displayed form through whole-cell patch-clamp assays on human T cells. Phage concentrations for comparative testing were guided by the relative effectiveness of phage binding to T+F chimeras (see Fig. 6*D*). The phage concentrations needed for an approximate 50% block of the current were experimentally titrated for all three phage-conjugated toxins (Figs. 9, *A* and *B*, and S4, *A*–*C*). By using this approach, the affinity of the phage-displayed peptides for Kv1.3 is inversely proportional to the phage concentration required to block the current by 50%, that is, lower inhibitory phage concentration indicates higher affinity. All three tested peptides exerted significant inhibition of the Kv1.3 current in their phage-displayed form, showing that C-terminal fusion of these toxins to the P3 envelope protein of M13 phages does not hinder their inhibitory potential. The estimated relative potencies calculated from the phage concentrations required to reach 50% blocking effect were approximately 1, 1/30, and <1/600 for Vm24, HgTx1, and KTX1, respectively (Fig. 9*B*). These relative potencies were in accordance with the published potencies of native peptide forms, as indicated by IC_50_ values of 3 pM, 86 pM, and 650 pM, respectively (Table 1) (16, 47, 48).

Validity of these latter electrophysiological measurements was also supported by the fact that tetraethylammonium (TEA^+^) at a concentration of 10 mM, used as a positive control, caused approximately 50% inhibition of Kv1.3, indicating the complete exchange of the recording solutions around the cells. Moreover, the previously characterized mutant of KTX1 - DDDKTX1, that is unable to bind to the Kv1.3 channel (20), did not block Kv1.3 currents in phage-displayed form, supporting validity of our patch-clamp measurements as well (Fig. S4*D*). This served as an important negative control to exclude the possibility that the phage construct by itself could inhibit the hv1.3 current, independently of the fused toxin.

## Discussion

In this study, we aimed to develop improved target proteins for drug discovery, focusing on the optimization of a solid-phase–based test system to find potential Kv1.3 channel blockers. Through heterologous recombinant expression, we generated modified KcsA-Kv1.x chimeras with targeted amino acid mutations not only in the turret (T) but also in the filter (F) regions.

Legros *et al.* (34) showed that *E. coli* can be employed as an expression host to produce active chimeric KcsA protein harboring the turret region of the human Kv1.3 channel. However, attempts to produce active chimeras containing both the turret and filter region (T+F) of human Kv1.3 channels in a heterologous expression system proved challenging, due to weak expression and improper assembly of monomers resulting in defective tetramers (30, 34). Therefore, further attempts to generate chimeras focused on transferring only the turret region of different Kv1.x proteins into KcsA protein (35, 50, 51, 52). In our work, we used a bacterial expression system as a simple and cost-effective approach to generate sufficient amount of recombinant KcsA-Kv1.x T+F chimeras. To improve protein expression, we systematically tested the effect of several additives, such as potassium chloride, glycerol, and glucose (data not shown). We investigated several parameters that can have positive influence on protein yield as well (inducer concentration, induction temperature, and time, not shown). However, none of the aforementioned factors had such a significant impact on the expression level of active chimeras as the expression strain itself. Although we obtained substantial amounts of re-solubilized proteins extracted from the membrane fractions of several commercially available *E. coli* strains, only the C41(DE3) strain produced active tetramers (53). The C41(DE3)—a derivative of the parental BL21(DE3) strain—is widely used to produce toxic or membrane proteins mainly due to the weakened T7 RNAP activity resulted from mutations in the lacUV5 promoter (54). Slowing the expression of target proteins might aid the correct membrane assembly of monomers yielding active tetrameric channels.

Using our improved expression protocol, we prepared a significant amount of Kv1.1 and Kv1.3 chimeras; however, the production of both the T-only and T+F Kv1.2 chimeras proved to be challenging. Others also reported poor yield of the Kv1.2 T-only chimera (35). One explanation could be that the correct cell membrane assembly of the Kv1.2 chimeras is seriously limited, due to the amino acid composition of the turret region, resulting in accumulation of the expressed protein in inclusion bodies. In addition, it seems that the purified Kv1.2 chimeras are less stable compared to the other four chimeras (T-only and T+F chimeras of KcsA-Kv1.1 and -Kv1.3) as it was indicated by SDS-PAGE analysis. It might require tremendous effort and time to improve protein yield further; however, the produced material was enough to conduct subsequent experimental applications such as phage ELISA and competition assays. Importantly, the quantity and quality of produced protein is sufficient to support multiple HTS campaigns in the future.

Several binding assays were applied in the past to investigate channel protein and ligand interactions, such as fluorescent and radioligand-based techniques. One of the earliest approaches to study interactions between recombinantly produced channel proteins and ligands required the immobilization of the target protein onto an adhesive surface such as CM5-dextrane; however, it also required the use of radioligands (30, 44, 55). A more convenient method is the traditional phage ELISA assay, in which the target protein is immobilized onto protein-binding 96-well polystyrene plates (such as MaxiSorp), and the toxin peptides are presented on the surface of M13 phages (56). Solid phase biopanning of phage libraries conducted on MaxiSorp plates yielded high affinity binders of Kv1.3 (20). Due to the low immobilization efficiency of T-only and T+F chimeras on MaxiSorp plates, we had to improve the plate-binding assay protocol by immobilizing His-tagged chimera proteins on nickel-coated plates. It turned out that this was a more effective approach yielding more intensive and reliable binding results with much lower concentrations of the target protein, probably due to a more ordered immobilization leaving the majority of the binding sites (turret and filter regions) of the tetrameric channel proteins accessible.

As we established reliable phage ELISA protocols to assess ligand-binding interactions, we compared toxin-binding patterns (rank order of affinities) of the T-only and T+F chimera variants of Kv1.1, Kv1.2, and Kv1.3 channels to the respective channel-blocking potencies. Our results clearly showed that the ligand-binding patterns of Kv1.2 and Kv1.3 T-only chimera constructs were strikingly different from the potency patterns on the corresponding native channels. Inclusion of both the turret and the correct filter regions in these chimeras (T+F versions) restored the order of affinities to be concordant with the order of native channel-blocking potencies. However, in the case of Kv1.1, both T-only and T+F chimeras appeared to represent well the membrane-embedded human potassium channels in solid phase assays. It should also be noted that results of phage ELISA and competition assays on the same type of chimeras, be it either T-only or T+F, were concordant irrespective of whether the toxins were tested in a native or in a phage-displayed form. These encouraging results suggest that measurements with toxins expressed on phages can be predictive for the binding and blocking behavior of the corresponding drug substances. This was further supported by the observation that the selected toxins expressed on phages could block Kv1.3 channels of human lymphocytes with apparently similar relative potencies to published results obtained with native toxins.

It was intriguing to observe that ligand-binding patterns of Kv1.1 T-only and T+F as well as Kv1.2 T-only chimeras were all similar to that of the native Kv1.1, showing a rank order of affinities ShK > KTX1 > MTX. MTX, a potent blocker of the native Kv1.2 channel, completely lost its affinity to the Kv1.2 T-only as compared to the T+F chimera (Fig. 7). The Kv1.2 T-only differs from the T+F chimera in three amino acids mutations: M81L, V82Y, and T84V (see sequences in Fig. 1*B*). Thus, the radical conversion of the behavior of KcsA-Kv1.2 T-only to a Kv1.1-like might be attributed to these residues. Methionine to leucine mutation at position 81 between the KcsA-Kv1.1 T-only and T+F variants did not alter their binding profile. Significant impact of the T84V mutation is also unlikely, since it is probably inaccessible for ligand binding as deposited Kv1.2 structures in the Protein Data Bank suggest (PDB, (57, 58, 59). Moreover, the strong binding of MTX-expressing phages to a T+F chimera variant did not diminish when T84 was mutated in a pilot experiment (not shown). Therefore, most probably, the valine to tyrosine mutation in position 82 (corresponding to position 381 in the human Kv1.2 protein) caused the dramatic loss of MTX binding and enhancement of the KTX1 and ShK binding in the case of T-only KcsA-Kv1.2 chimera. This notion is also supported by the studies of Visan *et al.* (36) (2004) based on mutation analysis and electrophysiological measurements, concluding that valine in this position is responsible to mediate subtype-specific target–ligand interactions. The structure of the complex of the so-called Kv1.2-paddle chimera and a natural peptide toxin, charybdotoxin, further supports the importance of valine at this position. In this complex, the aromatic ring of Tyr36 from the peptide is positioned to pack against Val377 (corresponding to Val381 in the human Kv1.2) (60).

The difference between the ligand-binding profiles of KcsA-Kv1.3 T-only and T+F chimeras, with the latter representing well the native Kv1.3 channel protein, might be attributed to mutations M81L and H82Y (positions 450 and 451 in the human Kv1.3 channel; see sequences in Fig. 1*B*). We hypothesize that since the M81L mutation had little impact on Kv1.1 chimeras (see above), replacement of histidine by tyrosine could be responsible for the drastic difference in the ligand-binding profiles of the Kv1.3 T-only and T+F chimeras. This notion is in line with structural data obtained from single particle cryo-EM of the complex of monovalent Fab-ShK-human Kv1.3 channel (61). Serine 20 and arginine 11 of ShK form hydrogen bonds with His451 on separate subunits of the channel. Molecular docking studies involving Vm24-toxin have also revealed that the E11 residue of the toxin interacts with His451 of the human Kv1.3 channel (40).

In conclusion, we successfully produced recombinant KcsA-Kv1.1, KcsA-Kv1.2, and KcsA-Kv1.3 chimeric proteins in which the amino acid sequences of both the turret and filter regions were changed to their corresponding segments of the human Kv1.x channel proteins. Phage-binding and competition studies conducted with our new chimeras in improved solid-phase assays were more predictive for electrophysiological channel-blocking potencies. These chimeras can help the discovery and optimization of novel potent and selective Kv1.3 blocking drugs that might be useful for better treatment of autoimmune diseases. Our results also provide valuable information about structural requirements of target–ligand interactions of the tested Kv.1.x channels and channel-blocking ligands.

## Experimental procedures

### Toxin test substances

The tested synthetically produced peptide toxins [KTX1 (STK-370), HgTX1 (STH-400), ShK (STS-400), Vm24 (STV-055), MTX (STM-340)] were purchased from Alomone Labs. Peptide toxins were dissolved in USP water to 100 μM concentration, then aliquoted, and stored at −20 °C.

### Cloning of WT KcsA and KcsA-Kv1.x chimeras

The coding sequence of full-length WT KcsA (UniProtKB/Swiss-Prot: P0A334.1) and the KcsA-Kv1.3 T-only chimeric construct were cloned into a pQE30 vector between BamHI and PstI restriction sites. Both constructs harbored an N-terminal His-tag for the purpose of downstream purification processes. The coding sequences of the KcsA-Kv1.1, 1.2 T-only and T+F chimeras, and the KcsA-Kv1.3 T+F construct were cloned into a pET45b expression plasmid between KpnI and HindIII restriction sites. These expression constructs also harbored an N-terminal 6xHis-tag.

### Expression of WT KcsA and KcsA-Kv1.x chimeric proteins

pQE30 expression plasmids harboring the coding sequences of the full-length WT KcsA channel protein or the Kv1.3 T-only chimera were transformed into XL1-Blue *E. coli* cells (2022249, Agilent) and streaked onto Luria-Bertani (LB) plates supplemented with 100 μg/ml carbenicillin and 0.2% v/v glucose. The plate was incubated overnight at 37 °C in a stationary incubator. Several colonies were inoculated into 50 ml LB starter media supplemented with 100 μg/ml carbenicillin and 0.2% v/v glucose. The cell culture was incubated in a shaker incubator at 30 °C overnight at 200 rpm, and the next day, 5 to 5 ml starter culture was inoculated into 0.5 to 0.5-l LB media supplemented with 100 μg/ml carbenicillin, 0.2% v/v glucose, and 0.4% glycerol and cultivated at 37 °C at 200 rpm. When reaching midlog phase (A600: 0.6), the cell culture temperature was tapered to 30 °C, 5 mM BaCl_2_ was added, then the cultures were induced for 3 h with 1 mM IPTG (437145X, VWR Chemicals). Cells were pelleted by centrifugation at 5000*g* for 10 min and stored at −20 °C.

pET45b plasmids harboring Kv1.x T-only and T+F chimeric constructs were transformed into C41(DE3) *E. coli* competent cells (60442-1, Lucigen), then streaked onto LB plates supplemented with 100 μg/ml carbenicillin and 0.2% v/v glucose. The plate was incubated overnight at 37 °C in a stationary incubator. Several colonies were inoculated into 50 ml LB starter media supplemented with 100 μg/ml carbenicillin and 0.2% v/v glucose. The cell culture was incubated in a shaker incubator at 30 °C overnight at 200 rpm, and the next day, 5 to 5 ml starter culture was inoculated into 0.5 to 0.5-L LB media supplemented with 100 μg/ml carbenicillin, 0.2% v/v glucose, and 0.4% glycerol and cultivated at 37 °C at 200 rpm. When reaching midlog phase (A600: 0.6), the cell culture’s temperature was tapered to 30 °C, then 5 mM BaCl_2_ was added, and the cultures were induced for overnight with 0.1 mM IPTG at 18 °C. Cells were pelleted by centrifugation at 5000*g* for 10 min and stored at −20 °C.

### Purification of WT KcsA and KcsA-Kv1.x chimeric proteins

Frozen cell pellet was resuspended in ice-cold lysis buffer (50 mM Tris, pH 8.0, 300 mM KCl, 5% glycerol) supplemented with SIGMA*FAST* protease inhibitor (S8820, Sigma-Aldrich), then lysed by ultrasound followed by pelleting the cell debris at 18,000*g* for 15 min. The supernatant was subjected to ultracentrifugation with 100.000*g* for 1 h at 4 °C. The pelleted membrane fraction was resolubilized in resolubilization buffer (50 mM Tris, pH 8.0, 300 mM KCl, 5 mM imidazole) supplemented with 20 mM DDM (850520P-1G, Avanti). Resolubilized material was ultracentrifuged at 100.000*g* for 1 h, and the supernatant was loaded onto a HiTrap TALON Crude chromatographic column (29-0485-65, Cytiva) pre-equilibrated with washing buffer (50 mM Tris pH 8.0, 300 mM KCl, 2 mM DDM, 5 mM imidazole) and connected to an Akta Pure chromatographic device. The loaded column was washed with washing puffer, then bound material was eluted with a linear gradient (0–100%, in 10 column volume) of elution buffer (50 mM Tris pH 8.0, 300 mM KCl, 2 mM DDM, 250 mM imidazole) in 1 ml elution fractions. Fractions were analyzed by SDS-PAGE.

Fractions containing the protein of interest were pooled and loaded onto a HiLoad 16/600 Superdex 200 pg gel filtration column (28-9893-35, Cytiva), pre-equilibrated with gel filtration/storage buffer (20 mM Tris pH 8.0, 300 mM KCl, 2 mM DDM) and connected to an Akta Pure chromatographic device. Elution was done at 1 ml/min flow rate. Eluted peak fractions (1 ml) were analyzed by SDS-PAGE, then pooled and concentrated. The concentration of the purified protein was determined by measuring the UV absorbance at 280 nm using the protein extinction coefficient calculator in the ProtParam tool of Expasy (https://web.expasy.org/protparam/). Then the protein samples were aliquoted, flash-frozen, then stored at −80 °C. To assess activity, chimeric proteins were subjected to phage ELISA using a dilution series of M13 bacteriophage displaying HgTx1, which inhibits with equivalent high potency Kv1.1, Kv1.2, and Kv1.3 channels (47). The applied phage concentrations were ranging between 10^9^ and 10^11^ particle/ml.

### Western blot analysis of expressed proteins

For western blot analysis, 0.5 to 0.5 μg protein sample/well were loaded onto commercial 4 to 20% Novex Tris-Glycine SDS gel (XP04205BOX, Invitrogen) and were ran at 200 V for 55 min. Proteins were transferred onto nitrocellulose membrane using an XCell II Blot Module apparatus (Invitrogen) at 25 V for 90 min. The protein transfer was inspected by Ponceau S (A40000279, Thermo Fisher Scientific) staining, then the membrane was blocked in 3% low fat milk powder in TBS (Tris-based saline) for 1 h, then incubated in mouse 6×-His tag monoclonal antibody (4E3D10H2/E3, Invitrogen) diluted 1/2500 in 3% low fat milk powder in TBS for 1 h. The membrane was washed with 3 × 5 min in TBS-0.1% Tween, then was incubated for 1 h in secondary Goat anti-mouse IgG HRP-conjugated antibody (31430, Invitrogen) diluted in 1: 5000 in TBS for 30 min. The membrane was washed 3 × 5 min in TBS, then Pierce 1-Step Ultra TMB Blotting Solution (37574, Thermo Fisher Scientific) was added. The color development was stopped at 5 min by rinsing the membrane with ultra-pure water.

### Native gel electrophoresis

0.5 to 0.5 μg protein sample/well were loaded onto commercial NativePAGE 4 to 16%, Bis-Tris, Mini Protein Gel (BN1002BOX, Invitrogen) and were ran at 150 V, 110 min using the NativePAGE Sample kit (BN2008, Invitrogen). The gel was destained in 8% v/v acetic acid.

### Cells for patch-clamp recordings

Chinese Hamster Ovarian cells were grown in Dulbecco’s modified Eagle’s medium–high glucose supplemented with 10% FBS, 2 mM L-glutamine, 100 U/ml penicillin-g, and 100 μg/ml streptomycin (Invitrogen) at 37 °C in a 5% CO_2_ and 95% air humidified atmosphere. Cells were passaged twice per week following a 5 min incubation in PBS containing 0.2 g/L EDTA. Chinese Hamster Ovarian cells were transiently transfected with plasmids encoding human Kv1.1 (*hKCNA1* gene) and human Kv1.2 (*hKCNA2* gene) in pCMV6-AC-GFP plasmid (RG211000 and RC222200, OriGene Technologies) using Lipofectamine 2000 (Invitrogen) following the manufacturer’s protocol, then cultured under standard conditions. Transfected cells were washed twice with 2 ml of extracellular solution (ECS, see below) and replated onto 35 mm polystyrene cell culture dishes (Cellstar, Greiner Bio-One) before the patch-clamp experiments. At 24 h after transfection, GFP-expressing transfectants were identified with Nikon TE 2000U fluorescence microscope (Nikon) using bandpass filters of 455 to 495 nm and 515 to 555 nm for excitation and emission, respectively, and used for current recordings (∼60–70% success rate for co-transfection). In general, currents were recorded 24 to 36 h after transfection.

Human Kv1.3 currents were recorded from activated peripheral blood mononuclear cells (see below) 3 to 4 days after activation. Heparinized human peripheral venous blood was obtained from healthy volunteers. Mononuclear cells were separated through Histopaque-1077 density gradient centrifugation. Collected cells were washed twice with Ca^2+^- and Mg^2+^-free Hanks’ solution containing 25 mM Hepes buffer, pH 7.4. Cells were cultured for 3 to 4 days in 24-well culture plates in a 5% CO_2_ incubator at 37 °C, in RPMI 1640 medium supplemented with 10% fetal calf serum (Sigma-Aldrich), 100 μg/ml penicillin, 100 μg/ml streptomycin, and 2 mM L-glutamine (density, 0.5 × 106 cells per ml). Phytohemagglutinin A (Sigma-Aldrich) was added to the medium at 10 μg/ml to amplify the Kv1.3 expression. Cells were washed gently twice with 2 ml of ECS for the patch-clamp experiments.

### Patch-clamp assay

Conventional whole-cell patch-clamp electrophysiology was used to record ionic currents. Micropipettes were pulled from GC150F-7.5 borosilicate capillaries (Harvard Apparatus) with tip diameters between 0.5 and 1 μm resulting in a tip resistance of 2 to 8 MΩ in the extracellular (bath) solution. All measurements were carried out by using Axopatch 200B amplifier connected to a personal computer using Axon Digidata 1550A data acquisition hardware and Pclamp10.7 software (https://support.moleculardevices.com/s/article/Axon-pCLAMP-10-Electrophysiology-Data-Acquisition-Analysis-Software-Download-Page). The holding potential was −120 mV. Records were discarded when leak at the holding potential was >10% of peak current at the test potential. Experiments were performed at room temperature (20–24 °C). Before analysis, whole-cell current traces were corrected for ohmic leakage and digitally filtered with a three-point boxcar smoothing filter.

Solutions: all salts and components of the solutions were purchased from Sigma Aldrich. The extracellular (bath) solution (ECS) contained 145 mM NaCl, 5 mM KCl, 2.5 mM CaCl_2_, 1 mM MgCl_2_, 10 mM Hepes, and 5.5 mM glucose (pH 7.35 with NaOH), while the intracellular (pipette) solution (ICS) contained 140 mM KF, 2 mM MgCl2, 1 mM CaCl2, 11 mM EGTA, and 10 mM Hepes (pH 7.22 with KOH). The osmolarity of the ECS and ICS were 302 mOsM.

Native peptides, phage-expressed peptides, and positive controls used for them were dissolved in ECS supplemented with 0.1 mg/ml bovine serum albumin (BSA; Sigma-Aldrich Hungary). Bath perfusion around the measured cell with different extracellular solutions was achieved using a gravity flow micro perfusion system using a rate of 0.5 ml/min. Excess fluid was removed continuously.

Voltage protocols: For measurement of Kv1.1 to 1.3 currents, voltage steps to +50 mV were applied from a holding potential of −120 mV and the peak current was measured every 15 s. For Kv1.3 currents, 15-ms-long depolarizing pulses were applied. For Kv1.1, 50-ms-long–activating stimuli were used. Kv1.2 currents were evoked by 200-ms-long pulses. Positive controls were applied at a concentration equivalent to their IC_50_ values (0.3 mM and 10 mM TEA+ for Kv1.1 and Kv1.3, respectively, and 14 nM charybdotoxin for Kv1.2). The remaining current fraction at a given molar concentration was calculated as I/I_0_, where I_0_ is the peak current in the absence and I is the peak current at equilibrium block or in the absence of inhibition after ∼2 min perfusion by the investigated peptide/phage.

### Preparation of phages for phage-displayed peptide studies

Phage display test solutions were prepared according to routine phage display protocols (62). Phosphorylated and annealed oligonucleotides encoding the peptide construct were ligated into linearized pAS62 phagemid vector (https://patents.google.com/patent/WO2022144560A1/en) at a vector/insert ratio of 1:3. The final phagemid construct harbored a signal sequence followed by the peptide of interest, a linker sequence (GSASSATR), and the C-terminal part of the P3 coat protein (amino acids: 216-424 of NP_510891.1). Chemically competent XL1-Blue *E. coli* cells (2022249, Agilent) were transformed with the ligation product and then streaked onto ampicillin (100 μg/ml) containing LB/Amp plates and incubated for 16 h at 37 °C. A few clones were sequenced, and a single colony with confirmed target sequence was inoculated into 3 ml 2 YT medium supplemented with 100 μg/ml ampicillin and incubated at 37 °C until the culture reached midlog phase. The culture was infected with M13KO7 helper phage (N0315S, New England Biolabs) (phage/cell ratio 10:1) and was incubated for 30 min at 37 °C. The 3 ml culture was transferred into 200 ml 2YT medium, supplemented with 100 μg/ml ampicillin and 25 μg/ml kanamycin, and incubated overnight at 37 °C. The overnight cell culture (200 ml) was pelleted by centrifugation at 8000*g* for 10 min in 400 ml centrifuge tubes at 4 °C. The supernatant was transferred into a fresh 400 ml centrifuge tube containing 40 ml PEG/NaCl (200 g/l PEG-8000 and 146.1 g/l NaCl) and then was mixed thoroughly and incubated at room temperature for 20 min. Phages were pelleted by centrifugation at 18,000*g* for 15 min at room temperature. After discarding the supernatant, the tubes were centrifuged again at 1000 rpm for 1 min to collect and remove any residual media, then phages were solubilized in 4 ml phage resuspension buffer (TBS containing 0.5% BSA and 0.05% (v/v) Tween-20), and all insoluble materials were removed by centrifugation at 18,000*g* for 10 min at 4 °C. The supernatant was transferred into four 1.5 ml centrifuge tubes (4 × 1 ml) and precipitated again with 100 to 200 μl PEG/NaCl and was incubated for an additional 5 min before centrifuged again at 12,000*g*. The pellets were resuspended in low volumes (150–300 μl) of phage resuspension buffer and combined into a single phage solution. The particle concentrations of phage solutions were determined using a NanoDrop One C (Thermo Fisher Scientific) spectrophotometer. The phage particle concentration was assessed according to the formula: (A_268_–A_320_) × 5 × 10^12^ particles/ml (62). Phage stock solution stored at 4 °C.

### Phage ELISA-binding assay

Phage stock solutions were diluted to working concentration with phage resuspension buffer supplemented with 2 mM DDM. For protein immobilization, the following amounts were used in a final volume of 50 μl/well: wt KcsA, 0.1 μg; KcsA-Kv1.1 T-only, 0.005 μg; KcsA-Kv1.1 T+F, 0.02 μg; KcsA-Kv1.2 T-only, 0.01 μg; KcsA-Kv1.2 T+F, 0.1 μg; KcsA-Kv1.3 T-only, 0.01 μg; and KcsA-Kv1.3 T+F, 0.1 μg. Protein samples were diluted in 50 μl of storage buffer (20 mM Tris pH8.0, 300 mM KCl, 2 mM DDM) and were added to the designated wells of a Pierce Clear, 96-Well Nickel Coated Plate (15442, Thermo Fisher Scientific), then incubated for 1 h at room temperature on a horizontal shaker at 130 rpm. The plate was blocked for 1 h at room temperature with 180 μl/well TBS-BSA supplemented with 1 mM DDM. Following blocking, the wells were washed 4× with washing buffer (300 μl TBS-Tween, 0.1% v/v) using a Wellwash Versa microplate washer (Thermo Fisher Scientific); then 50 μl phage solution (in TBS-BSA-Tween, 0.1% v/v, 2 mM DDM) at determined phage concentration was added to the wells and incubated for 1 h at room temperature on a horizontal shaker. The plate was washed 4× with washing buffer, then 50 μl anti-M13 IgG HRP (MA5-36125, Invitrogen) diluted to 1:2500 in TBS-Tween (0.1% v/v) was added to the wells and incubated for 1 h at room temperature. The plate was washed again 4× with 300 μl/well washing buffer. Finally, 50 μl/well 1-Step Ultra TMB-ELISA solution (34028, Thermo Fisher Scientific) was added to the wells, and the developing signal was stopped with 50 μl 1M HCl after 5 to 10 min. The light absorbance signal was read at 450 nm with Byonoy absorbance 96 plate reader (Byonoy GmbH).

### Phage-binding competition assay

For protein immobilization, the following amounts were used in a final volume of 50 μl/well: KcsA-Kv1.1 T-only, 0.005 μg; KcsA-Kv1.1 T+F, 0.01 μg; KcsA-Kv1.2 T-only, 0.025 μg; KcsA-Kv1.2 T+F, 0.0125 μg; KcsA-Kv1.3 T-only, 0.01 μg; and KcsA-Kv1.3 T+F, 0.1 μg. Protein samples were diluted in storage buffer. Binding of given concentrations of relevant ligand-displaying phages quantified by light absorbance (A 405 nm) were investigated in a similar manner as in phage ELISA-binding assay, while different concentrations of competing toxin test substances were applied simultaneously. The results were converted to percent inhibitions (percent displacement) of the indicator phage binding, and relative affinities of the tested toxins were assessed by plotting percent displacements against log concentrations and determining half maximal inhibitory concentrations (IC_50_) by sigmoidal curve fitting using “Sigmoidal dose-response (variable slope) with least squares method” algorithm in GraphPad Prism 8 using the following equation:

$$Y=\text{Bottom}+\frac{Top-Bottom}{{1+10}^{\left(LogEC50-\times \right)\cdot HillSlope}}$$

## Data availability

All main text data are in this manuscript. Supplemental data are in the corresponding Supporting Information document. Correspondence and requests for materials should be addressed to the corresponding author (peter.hornyak@vrgtherapeutics.com).

## Supporting information

This article contains supporting information.

## Conflict of interest

The authors declare that they have no conflicts of interest with the contents of this article.
