# Supplementary material for: KcsA-Kv1.x chimeras with complete ligand-binding sites provide improved predictivity for screening selective Kv1.x blockers
Source: J Biol Chem. 2024 Mar 11;300(4):107155. doi: 10.1016/j.jbc.2024.107155 (PMC11002876; doi:10.1016/j.jbc.2024.107155)
Supplement: Supporting Figures S1–S4 [file mmc1.docx]

**SUPPORTING INFORMATION**

KcsA-Kv1.x chimeras with complete ligand binding sites provide improved predictivity for screening selective Kv1.x blockers

Patrik Szekér, Tamás Bodó, Katalin Klima, Ágota Csóti, Nikoletta Ngo Hanh, József Murányi, Anna Hajdara, Tibor Gábor Szántó, György Panyi, Márton Megyeri, Zalán Péterfi, Sándor Farkas, Norbert Gyöngyösi and Péter Hornyák

**Patch-clamp assay**

Conventional whole-cell patch-clamp electrophysiology was used to record ionic currents. Micropipettes were pulled from GC150F-7.5 borosilicate capillaries (Harvard Apparatus, Kent, UK) with tip diameters between 0.5 and 1 μm resulting in a tip resistance of 2–8 MΩ in the extracellular (bath) solution. All measurements were carried out by using Axopatch 200B amplifier connected to a personal computer using Axon Digidata 1550A data acquisition hardware and Pclamp10.7 software.


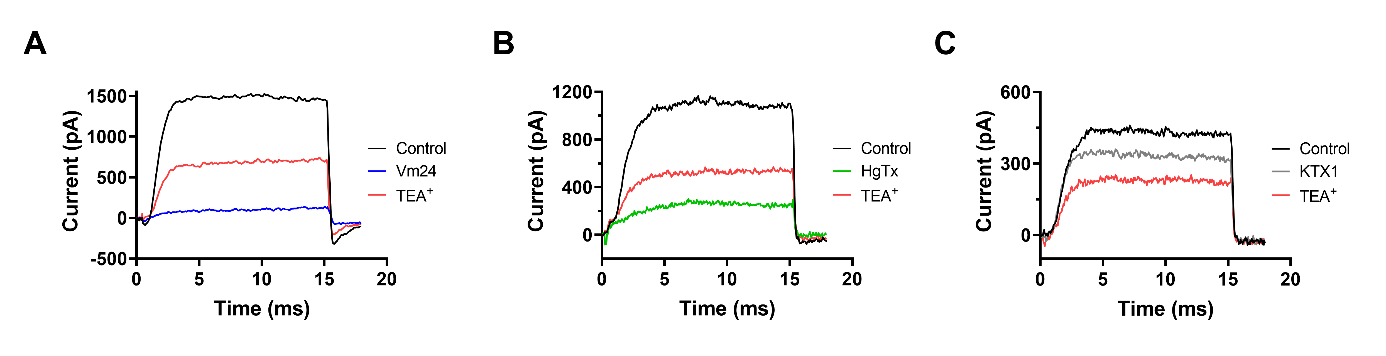


Fig. S1. Representative ionic current images of the blocking effect of native peptide toxins on the hKv1.3 channel. *A*, Blocking effect of Vm24 (trace marked with blue). *B*, Blocking effect of HgTx (trace marked with green). *C*, Blocking effect of KTX1 (trace marked with grey). The perfusion concentration of peptide toxins was 0.1nM. As a positive control, TEA+ was used at 10 mM (trace marked with red in all images). Abbreviations: Vm24: Vm24 toxin; HgTx1: Hongotoxin-1; KTX1: Kaliotoxin-1; TEA+: tetraethylammonium.


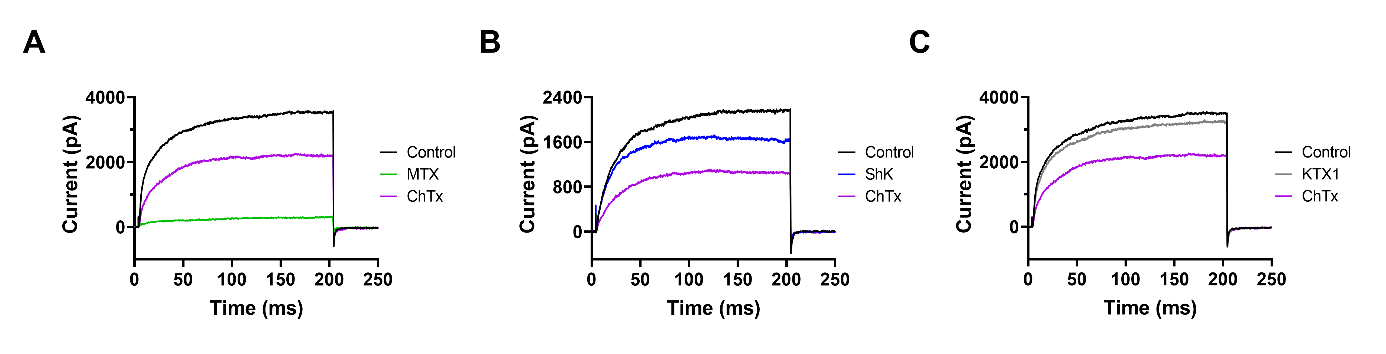


Fig. S2. Representative ionic current images of the blocking effect of native peptide toxins on the hKv1.2 channel. *A*, Blocking effect of MTX (trace marked with green). *B*, Blocking effect of ShK (trace marked with blue). *C*, Blocking effect of KTX1 (trace marked with grey). The perfusion concentration of peptide toxins was 10 nM. As a positive control, ChTx was used at 14 nM (trace marked with magenta in all images). Abbreviations: MTX: Maurotoxin; ShK: ShK toxin; KTX1: Kaliotoxin-1; ChTx: Charybdotoxin.


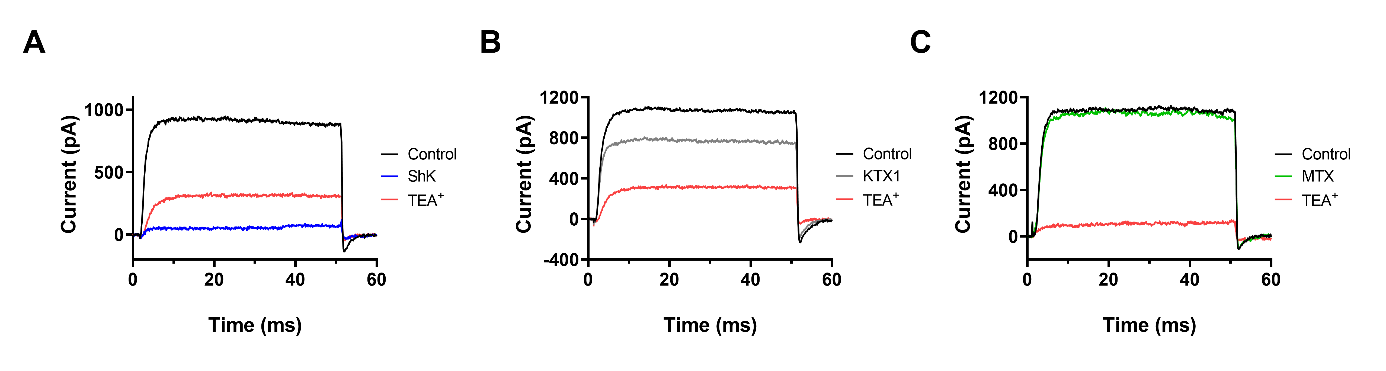


Fig. S3. Representative ionic current images of the blocking effect of native peptide toxins on the hKv1.1 channel. *A*, Blocking effect of ShK (trace marked with blue). *B*, Blocking effect of KTX1 (trace marked with grey). *C*, Blocking effect of MTX (trace marked with green). The perfusion concentration of peptide toxins was 1 nM. As a positive control, TEA+ was used at 0.3 mM (trace marked with red in all images). Abbreviations: ShK: ShK toxin; KTX1: Kaliotoxin-1; MTX: Maurotoxin; TEA+: tetraethylammonium.


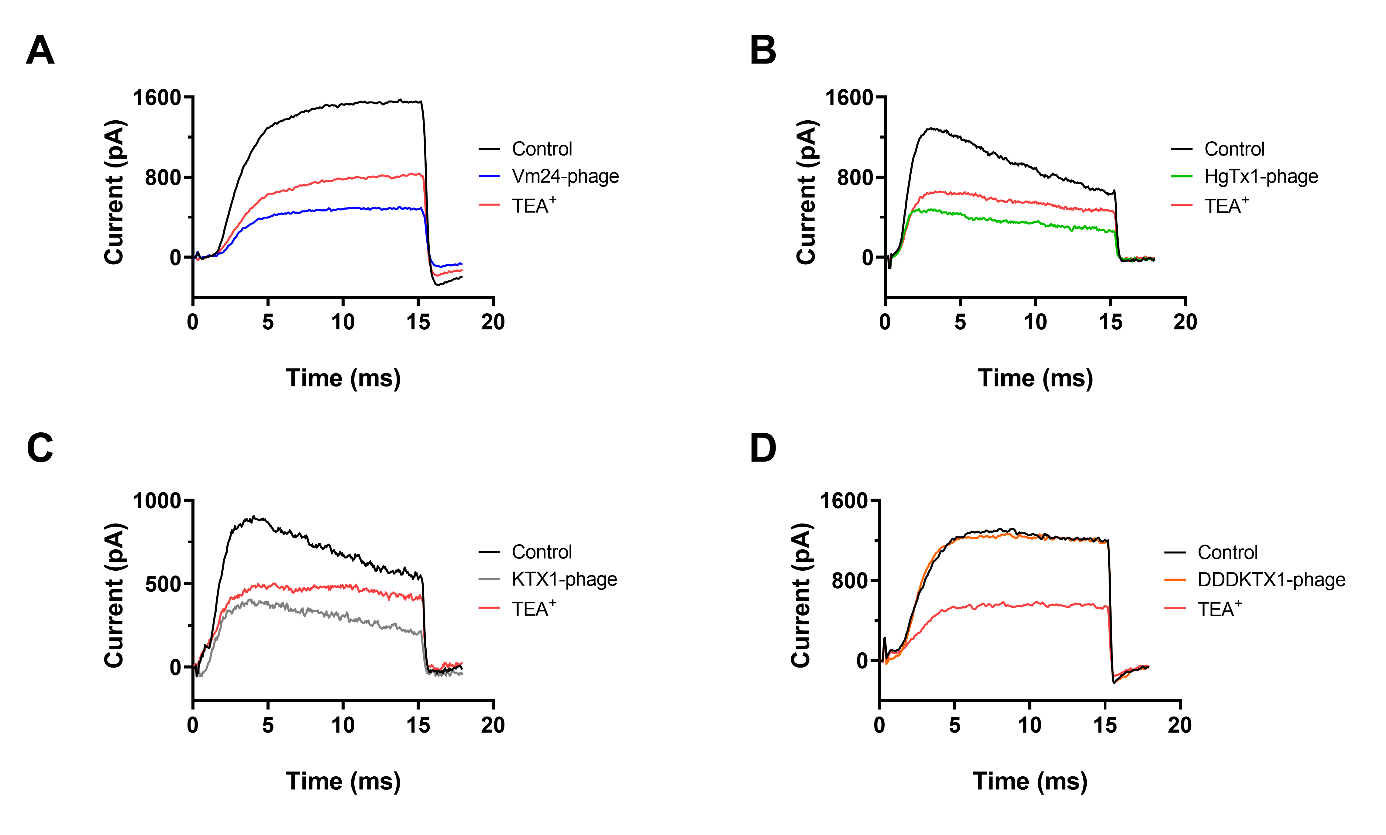


Fig. S4. Representative ionic current images of the blocking effect of phage displayed peptide toxins on the hKv1.3 channel. *A*, Blocking effect of Vm24-phages (trace marked with blue) at a perfusion concentration of 6 x 10^10^ particle/ml. *B*, Blocking effect of HgTx1-phages (trace marked with green) at a perfusion concentration of 1.8 x 10^12^ particle/ml. *C*, Blocking effect of KTX1 (trace marked with grey) at a perfusion concentration of 3.6 x 10^13^ particle/ml. *D*, As a negative control, DDDKTX1-phages (trace marked with orange) were used at a perfusion concentration of 3.6 x 10^13^ particle/ml. As a positive control, TEA+ was used at 10mM (trace marked with red in all images). Abbreviations: Vm24: Vm24 toxin; HgTx1: Hongotoxin-1; KTX1: Kaliotoxin-1; DDDKTX1: inactive mutant of Kaliotoxin-1; TEA+: tetraethylammonium.
